# Miscellaneous Selections

**Published:** 1855-10

**Authors:** 


### Miscellaneous Selections.

*Local Lesions of Fever.*—The most learned and celebrated physicians of France and Germany have found the principal local lesions of fever, to occur in the digestive organs. Latterly they have been almost confined to the mucous follicles of the small intestines—Pyer's glands—and names have been invented to indicate this as the peculiar anatomical characteristics of continental typhoid fever. Dr Stokes, however, says, he does not find this remarkable preponderance of lesions in the digestive organs, in Ireland, although an epidemic has prevailed there, "in which the condition of the intestines accurately represented that which is found to prevail on the continent." The local and secondary lesions of fever, are subject to still greater variations, perhaps in this country. The French and German peculiarity has not been ascertained to be extensively prevalent, the mucous lining of the colon and rectum being more commonly affected than that of the jejunum and ilium, giving rise to dysenteric complications; while the liver, stomach, and brain, are, perhaps, still more liable to become diseased. Of all the viscera, the only organs excused from implication are the lungs. A large and respectable class of physicians contend, that whenever pneumonic symptoms occur, the affection must always be considered as primary; and that pneumonia is not a secondary lesion of fever. Dr. Stokes is of a different opinion in reference to Irish typhus, in which pulmonary complications are common.

*Quinia as a Prophylactic.*—Besides the protection afforded American and English sailors, on the coast of Africa, from the fatal fevers of that region, it has been nearly proved, that quinia is the agent which will enable the civilized world to explore the hidden depths of the African continent, which have hitherto been considered almost inapproachable to the white man. Of thirty-eight men sent on the exploring expedition in 1805, under Mungo Park, only seven reached the Niger river, and finally all perished. Out of a still larger party under Captain Tuckey, in 1816, only one man returned alive. The brothers Lander, in 1832, lost forty Europeans out of forty-nine. And the better-appointed party under Capt. Buxton, in 1842 returned with forty-two men living out of one hundred and forty-two. But in 1852 the English government sent out a steamer to explore Tschadda, with sixty-six men, which ascended that river two hundred and fifty miles higher than it had been before explored, and returned without the loss of a single man. Something is due, perhaps, to the influence of the ordinary sanitary measures adopted, but we cannot suppose these to have been greatly superior to the pains-taking efforts of previous expeditions, and must attribute the unprecedented result to the influence of quinia, which we are told was used freely, both as a prophylactic and remedy.

*Eczema.*—Professor Benett, of Edinburg, recommends for the local treatment of this disease, keeping the affected part moist with lint or linen, saturated with a very weak alkaloid solution, consisting of half a dram of sub-carbonate of soda to a pint of water. This should be well covered over with oil-silk, or gutta-percha sheeting, to prevent evaporation. It soon relieves the irritation which is distressing to the patient, and prevents the formation of scabs. After a while the indurated parts become softened, the margins of the eruptions lose the fiery red color, and merge into that of the healthy skin. Finally, the whole surface assumes its normal character. He attaches great importance to the close adaptation of the dressings of the diseased surface and the constant moisture. He condemns the use of citrine ointment, and all other oily applications. Pemphigus, impetigo, ecthyma, and some other cutaneous eruptions, other than the exanthematæ, are treated in a similar manner, with alkaline solutions. No doubt much of the curative effect is due to the constant exclusions of the air from the diseased surface, to which Professor Bennett does not allude.

*Nitrate of Silver and Potassa.*—These two salts, by being melted together and cooled in moulds, form caustic pencils of any strength, dependent upon the proportion of potassa, and are generally made with a preponderance of two or three parts of the latter salt. They are used in cauterizing the eye-lids and eyes. If the part be washed immediately after application, with a solution of common salt, the nitrates are converted into chlorides, and may be washed away with water. This caustic is used, also, in gonorrhea, and other urethral and vaginal discharges, and in indolent and irritable ulcers. It is, of course, less expensive than common lunar caustic.

*Yellow Fever.*—Dr. Fenner, in the New Orleans *Medical News and Hospital Gazette*, contends, that the disease of the present season in that city is unquestionably of domestic origin; not a single case having been traced, by the remotest probability, to foreign infection. The hospital cases are brought in from different and remote parts of the city, showing no sort of communication whatever. All this can be well substantiated, and yet it is no more than has been proved a thousand times before, and it must not, therefore, be expected, that it will exert any special influence over the long disputed question of contagion and importation. The further remarks of Dr. F., which we quote below in his own words, are not less true and well established than his views above stated, and have indeed quite as important a bearing upon the mooted questions. In every city which has ever been visited by the disease, it has appeared and disappeared in precisely the way described, merging so gradually into other fevers both in its approach and decline, as to baffle the diagnostic skill of the most able and experienced physicians, no two of whom are likely to agree upon the line of demarkation between yellow, and common bilious fever; and thus proving, as far as human judgement can prove anything, that these diseases are essentially the same, differing only in degree of violence, and in the seat of the principal and most fatal local lesions. Dr. Fenner says:

"I maintain that this disease never *bursts out suddenly* here, presenting characteristic features different fron those of all other fevers, and therefore easily distinguishable by the experimental physician; but, on the contrary, that the endemic fevers (bilious, remittent and intermittent) gradually run into the yellow-fever type, assuming its features in such a manner as to excite *suspicion*, but yet not to justify a positive diagnosis, until one or more cases have been observed to terminate in black vomit or some other hemorrhage. Hence arise the discrepancies of opinion so often displayed by physicians as to the true character of certain cases that are seen at the beginning of an outbreak of yellow fever. At this period may be seen numerous cases of bilious remittent fever, presenting more or less the aspect of yellow fever, but not in so strong a light as to remove all doubt from the diagnosis.

"As the season advances, if it is going to be a *sickly one*, the yellow-fever type will become better marked and the number of cases will increase until it predominates over all others; then, if the disease prevails to a great extent, it is called an *epidemic*. This rages for a period usually, here, of sixty or seventy days, when it declines gradually and is again merged into the ordinary types of endemic fever.

"But if the season turns out to be *not very sickly*, the yellow type will not give the ascendancy over the others, but only appear *sporadically* and to a moderate extent. On these occasions we have plenty of intermittent and remittent fever, and some called *dengue*, which approach nearest the yellow-fever type, but few cases are marked yellow fever unless they present unquestionable signs."

*Preserving Ergot.*—With many others of the profession, we have had difficulty in preserving the medicinal power of ergot for any length of time; and perhaps it may be for the want of such preservation, that we find so much discrepancy in the statements made of its effects. When the patient is properly prepared for its action, by those relaxations favorable to uterine contractions and delivery, we have never found the pure article fail in its parturient power. These favorable conditions may often be secured by the anæsthetic influence of chloroform or ether. We have generally taken no other precaution in the preservation of ergot than to drop a few lumps of camphor into the bottle in which it is kept unpulverized; but Dr. Nunn, of Georgia, reports, in the American Journal of Pharmacy, a more certain and elegant method, by which the remedy can be preserved for several years. He reduces the ergot to a coarse powder by a common coffee-mill, and then carefully dries it. Then he prepares a solution of camphor in ether, eight grains to the ounce, a dram of which solution is placed in an ounce vial, which is immediately filled with the powdered ergot firmly packed, and tightly corked and sealed. The ergot is moistened throughout by the ether, which is evidence of the exclusion of air. The ether and camphor are dispelled by the heat of the water used in making an infuson for use. He has used ergot which had been preserved in this way for eight years without perceptible deterioration, and suggests, that other substances might be preserved upon the same principle, sustituting anhydrous alcohol for the ether when the latter is inadmissable. We venture to make the additional suggestion, that chloroform may, in many cases, be preferable to ether.

*New Source of Alcohol.*—"It is abstract, and not practical science, that is the life and soul of industry." This maxim of the celebrated Playfair has received an illustration by the formation of alcohol from coal gas. This consists, mainly, of bicarburetted hydrogen, forming what is called olefiant gas, which constitutes its principal illuminating power. The same is the basis of alcohol, which suggested to Bertholot the idea of converting it into this substance by uniting it with water. Accordingly it has been found, that by agitating it with sulphuric acid and metallic mercury the gas is absorbed, and upon adding water, and distilling the mixture, alcohol is obtained—the true ethylic alcohol, or spirit of wine. Thus is abstract science constantly leading to great results, whether it be in the discovery of a planet at the very place indicated by the astronomer, or in the synthetical production of alcohol in the precise manner pointed out by the chemist. And these are the triumphs of science founded on education, even in its present limited sphere.

*Causes of Headache.*—A German author divides the exciting causes of headache into three different kinds: Those directly affecting the brain itself, those proceeding from the digestive organs, and those derived from derangement of the sexual system. He has found a difficulty, as has every one else, in determining whether headache is dependent upon relation or emptiness of the intra-cranial vessels; and resorts to dry-cupping as a means of diagnosis. When applied to the nape of the neck, it will, in the one case, afford more or less immediate relief; in the other, it will increase the pain, and produce prostration and syncope. The inference is, that in the former instance the headache will be benefitted by diminishing the contents of the vessels; in the latter by increasing their amount. These remarks are no doubt just, but it must be borne in mind that depletion in anemia, and in cases of nervous debility and exhaustion, attended by pain and giddiness, sometimes gives temporary relief, although it must do ultimate injury. Therefore time must be allowed to determine the point. Quite as effectual means of diagnosis in these cases are found in stimulation, the beneficial and injurious effects of which are more prompt and decided. If a headache be relieved by brandy, by eating, or other means of excitation, an immediate inference may be drawn from it; and if it be increased by such experiments, the diagnosis is equally easy and certain.—*Memphis Med. Recorder.*

